# Association between serum calcium and in-hospital mortality in critically ill atrial fibrillation patients from the MIMIC IV database

**DOI:** 10.1038/s41598-024-79015-9

**Published:** 2024-11-14

**Authors:** Xin Zheng, Fenfang Zhang, Leigang Wang, Hongxuan Fan, Bing Yu, Xiaogang Qi, Bin Liang

**Affiliations:** 1 Department of Cardiology, Second Hospital of Shanxi Medical University, Taiyuan, Shanxi China; 2 Department of Cardiology, Yangquan First People’s Hospital, Yangquan, Shanxi China; 3 Orthopedics Department, Yangquan First People’s Hospital, Yangquan, Shanxi China

**Keywords:** Atrial fibrillation, Serum calcium, In-hospital mortality, Intensive care unit, Nonlinear, MIMIC-IV database, Biochemistry, Biomarkers, Cardiology, Diseases, Medical research, Risk factors

## Abstract

Thongprayoon et al. found in a study of 12,599 non-dialysis adult hospitalized patients that serum calcium (SC) disturbances affected more than half of the patients and were associated with increased in-hospital mortality. Similar impacts of SC disturbances on in-hospital mortality have been observed in patients with acute myocardial infarction and the general hospitalized population. Atrial fibrillation (AF), the most common arrhythmia in the intensive care unit (ICU), affects around 6% of critically ill patients. However, the significance of the relationship between SC levels and in-hospital mortality in these patients remains unclear. This study aimed to explore the correlation between SC levels and in-hospital mortality in ICU patients diagnosed with AF. Data from the MIMIC-IV database included 11,621 AF patients (average age 75.59 ± 11.74 years; 42.56% male), with an in-hospital mortality rate of 8.90%. A nonlinear relationship between SC levels and in-hospital mortality was observed. Effect sizes on either side of the inflection point were 0.79 (HR: 0.79, 95% CI 0.67–0.94, *P* = 0.006) and 1.12 (HR: 1.12, 95% CI 1.01–1.25, *P* = 0.029). Sensitivity analyses confirmed these results. SC levels around 8.56 mg/dL were associated with the lowest risk of in-hospital mortality, with risks increasing as SC levels deviated from this point. SC levels below this inflection point were linked to more pronounced clinical impacts. This finding has significant clinical implications for clinicians. Therefore, in the treatment of ICU patients with AF, clinicians should closely monitor SC levels, with a focus on maintaining them around 8.56 mg/dL.

## Introduction

Calcium, one of the most abundant minerals in the human body, plays essential roles in muscle function, nerve transmission, intracellular signaling, and vascular constriction and dilation regulation^1^. Additionally, intracellular calcium concentration changes are crucial in regulating cardiac contractility^2^. As a result, calcium levels are stringently controlled both cellularly and systemically, with variations affecting numerous physiological functions across various organs^3–5^.

In clinical settings, abnormalities in blood calcium and serum calcium (SC) fluctuations are notably prevalent among critically ill patients. These are influenced by several key factors, including magnesium levels, vitamin D, and parathyroid hormone (PTH)^6–9^. Both elevated and reduced SC levels are associated with adverse clinical outcomes, such as increased mortality, among hospitalized patients^10–14^. Notably, even after adjustments for age, sex, cardiovascular risk factors, echocardiographic parameters, and laboratory markers, low SC levels are linked to an elevated risk of postoperative Atrial fibrillation (AF) in patients undergoing isolated coronary artery bypass graft surgery, highlighting its significance in patients with AF^15^.

AF is the most common arrhythmia in the intensive care unit (ICU), affecting approximately 6% of critically ill patients^16^. It is particularly prevalent among those with specific conditions such as severe sepsis^17^. Not all patients with AF benefit from ICU admission, making it essential to consider the in-hospital mortality rate following ICU admission.

In a study by Thongprayoon, C. et al., involving 12,599 non-dialysis adult inpatients, it was found that serum ionized calcium disturbances affected over half of the patients and were associated with increased in-hospital mortality^18^. Similar effects of serum ionized calcium disturbances on in-hospital mortality have been observed in patients with acute myocardial infarction as well as in the general inpatient population^3,19^. To date, no studies have assessed whether SC serves as an effective predictor of in-hospital mortality in critically ill patients with AF. We analyzed data from the MIMIC-IV database concerning all AF patients admitted to the ICU to deepen our understanding of the relationship between SC levels and in-hospital mortality. Our goal was to determine the threshold at which deteriorating SC levels become a reliable predictor of in-hospital mortality in these patients.

## Methods

### Patients

**Fig. 1:**
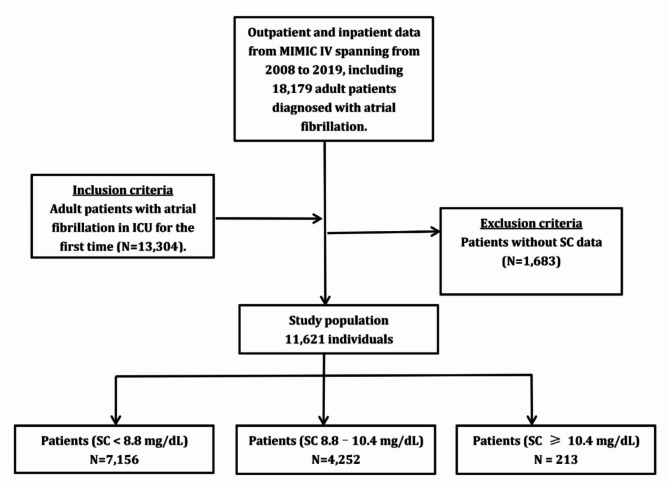
Flowchart of the study population. SC: serum calcium.

Patient data were collected from the publicly available MIMIC-IV database, which includes over 40,000 hospitalized patients admitted to the ICU at Beth Israel Deaconess Medical Center between 2008 and 2019. The inclusion criteria were adults (age ≥ 18 years) diagnosed with AF and admitted to the ICU for the first time. AF was identified using ICD-9 code 42,731 or ICD-10 codes I4891, I480, I482, I481, I4820, I4819, I4821, and I4811. Out of 18,179 AF patients, 13,304 were admitted to the ICU for the first time. The exclusion criteria applied to 1,683 patients who lacked SC data, leaving a final cohort of 11,621 AF patients ( Fig. 1). Patients were categorized into three groups based on normal adult SC levels (8.8–10.4 mg/dL): SC < 8.8 mg/dL (*n* = 7,156), 8.8 mg/dL ≤ SC < 10.4 mg/dL (*n* = 4,252), and SC ≥ 10.4 mg/dL (*n* = 213). Survival-related data were sourced from the ‘patients’ table, and information on hospital stay duration was obtained from the ‘admissions’ table^20^.

### Covariates

The baseline SC level was defined as the highest value recorded within the first 24 h after ICU admission, using data from the MIMIC-IV database. Normal adult SC levels generally range from 8.8 to 10.4 mg/dL. The primary objective of this study was to assess the in-hospital mortality rate following ICU admission. Participants included in the study were characterized by a variety of demographic and clinical variables, including age, sex, ethnicity, and medical conditions such as hypertension, heart failure, myocardial infarction (MI), stroke, obstructive sleep apnea syndrome (OSAS), and pneumonia. Conditions such as type 2 diabetes and type 1 diabetes were also considered, along with measurements like the lowest recorded levels of hemoglobin, potassium, and magnesium (Mg); the highest white blood cell (WBC) count; and levels of blood urea nitrogen (BUN), creatinine, glucose, and heart rate on the first day of ICU admission. Other factors evaluated included the first 24-hour Sequential Organ Failure Assessment (SOFA) score and the Simplified Acute Physiology Score II (SAPSII).

### Statistical methods

Continuous variables are presented as either the mean ± standard deviation (SD) or median and interquartile range (IQR), while categorical variables are expressed as frequencies or percentages. For the analysis of baseline characteristics, the Mann-Whitney test was used to compare continuous variables, and the chi-square test was utilized for categorical variables. Patients were categorized into three groups based on their SC levels (SC < 8.8 mg/dL, 8.8 mg/dL ≤ SC < 10.4 mg/dL, SC ≥ 10.4 mg/dL), aligning with the normal range of SC (8.8–10.4 mg/dL). To investigate the association between SC levels and in-hospital mortality following ICU admission, smooth curve fitting was employed, along with univariate and multivariate Cox regression analyses, utilizing both unadjusted and multivariate adjusted models to ensure the relationship’s stability. Regarding the selection of covariates, in Model I, we chose basic demographic characteristics. For Model II, covariates were added based on variables that showed a significant association with in-hospital mortality (*p* < 0.05) in the univariate regression analysis (Table 1), in addition to those used in Model I. In Model I, adjustments were made for age, sex, and ethnicity; Model II included adjustments for age, sex, ethnicity, hypertension, MI, OSAS, SOFA, SAPSII, WBC count, potassium, glucose, hemoglobin, BUN, creatinine, and heart rate. Results are reported as hazard ratios (HRs) with 95% confidence intervals (CIs). Threshold effect analyses were conducted to evaluate the predictive capability of SC levels for in-hospital mortality. All comparisons were pre-planned, and tests were two-sided, with *P* < 0.05 indicating statistical significance between two or more groups. All analyses were conducted using R version 4.3.2 (http://www.R-project.org, R Foundation) and Free Statistics version 1.9 (http://www.clinicalscientists.cn/freestatistics/)^21^.

**Table 1:** Associations between covariates and in-hospital mortality in patients with AF. AF: atrial fibrillation.

Variable	HR (95% CI)	*P* value
Age, y	1.03 (1.02,1.03)	< 0.001
Sex		
female vs. male	0.81 (0.71,0.91)	< 0.001
Ethnicity:		
black	Reference	
white	0.97 (0.72,1.30)	0.817
other	1.21 (0.89,1.64)	0.226
Hypertension, n (%)	0.84 (0.74,0.95)	0.007
Heart failure, n (%)	1.02 (0.90,1.15)	0.765
MI, n (%)	1.36 (1.14,1.63)	< 0.001
Stroke, n (%)	1.05 (0.88,1.27)	0.58
Pneumonia, n (%)	0.97 (0.86,1.10)	0.666
OSAS, n (%)	0.54 (0.42,0.69)	< 0.001
Type 2 diabetes, n (%)	0.98 (0.86,1.12)	0.766
Type 1 diabetes, n (%)	1.20 (0.64,2.23)	0.571
SOFA	1.12 (1.10,1.14)	< 0.001
SAPSII	1.04 (1.04,1.04)	< 0.001
Highest WBC (K/µL)	1.01 (1.00,1.01)	< 0.001
Lowest potassium (mEq/L)	1.41 (1.28,1.56)	< 0.001
Lowest Mg (mg/dL)	1.02 (0.89,1.18)	0.752
Highest glucose (mg/dL)	1.00 (1.00,1.00)	< 0.001
Lowest hemoglobin (g/dL)	1.04 (1.02,1.07)	0.002
Highest BUN (mmol/L)	1.01 (1.01,1.01)	< 0.001
Highest creatinine (mg/dL)	1.08 (1.04,1.11)	< 0.001
SC (mg/dL)	0.95 (0.88,1.02)	0.136
Highest heart rate (bpm)	1.00 (1.00,1.01)	< 0.001

### Ethical approval

This study was conducted in accordance with the guidelines set forth in the Declaration of Helsinki. It is a retrospective analysis using the MIMIC-IV database (version 2.2)^22^, which includes data from over 40,000 patients admitted to the ICUs of Beth Israel Deaconess Medical Center in Boston, MA, from 2008 to 2019. The database is accessible to individuals who have completed the Collaborative Institutional Training Initiative (CITI) program (Certification number 59607840 for Zheng). All patient information in the MIMIC-IV database was anonymized, and the requirement for informed consent was waived.

## Results

### Baseline characteristics of the study subjects

Within the MIMIC-IV database, a total of 11,621 patients with AF met the inclusion and exclusion criteria and were admitted to the ICU (Fig. 1). Table 2 presents the basic demographic characteristics of the participants, categorized by SC levels. The average age of the participants was 75.59 ± 11.74 years, with 4,946 (42.56%) being male. On the first day of ICU admission, the average SC level was 8.59 ± 0.78 mg/dL. The average SOFA score on the first day was4.91 ± 3.39, and the SAPSII score was 40.45 ± 13.01.

**Table 2:** Baseline demographic characteristics of the study population stratified by SC concentration. Notes: data are presented as the mean ± SD, median (IQR), or N (%). Abbreviations: SC: serum calcium; SD: standard deviation; IQR: interquartile range; MI: myocardial infarction; OSAS: obstructive sleep apnea syndrome; SOFA: sequential organ failure Assessment; SAPS II: simplified Acute Physiology score II; WBC: white blood cell; mg: magnesium; BUN: blood urea nitrogen.

Variables	Total (*N* = 11,621)	SC (mg/dL)			*P* value
		< 8.8 (*n* = 7,156)	8.8 ~ 10.4 (*n* = 4,252)	≥ 10.4 (*n* = 213)	
Age, y	75.59 ± 11.74	75.43 ± 11.70	75.98 ± 11.80	73.03 ± 11.66	< 0.001
Sex, n (%)					< 0.001
male	4946 (42.56)	2,870 (40.11)	1,959 (46.07)	117 (54.93)	
female	6675 (57.44)	4,286 (59.89)	2,293 (53.93)	96 (45.07)	
Ethnicity, n (%)					< 0.001
black	551 (4.74)	272 (3.80)	257 (6.04)	22 (10.33)	
white	8,542 (73.50)	5,297 (74.02)	3,117 (73.31)	128 (60.09)	
other	2,528 (21.75)	1,587 (22.18)	878 (20.65)	63 (29.58)	
Hypertension, n(%)	5,192 (44.68)	3,325 (46.46)	1,794 (42.19)	73 (34.27)	< 0.001
Heart failure, n(%)	5,243 (45.12)	2,962 (41.39)	2,181 (51.29)	100 (46.95)	< 0.001
MI, n (%)	1,042 (8.97)	593 (8.29)	433 (10.18)	16 (7.51)	0.002
Stroke, n (%)	1,478 (12.72)	851 (11.89)	597 (14.04)	30 (14.08)	0.003
Pneumonia, n (%)	3,258 (28.04)	2,032 (28.40)	1,160 (27.28)	66 (30.99)	0.276
OSAS, n (%)	1,184 (10.19)	687 (9.60)	477 (11.22)	20 (9.39)	0.020
Type 2 diabetes, n(%)	3,598 (30.96)	2,080 (29.07)	1,425 (33.51)	93 (43.66)	< 0.001
Type 1 diabetes, n(%)	76 (0.7)	41 (0.6)	33 (0.8)	2 (0.9)	0.274
SOFA	4.91 ± 3.39	5.03 ± 3.36	4.58 ± 3.34	7.31 ± 4.17	< 0.001
SAPSII	40.45 ± 13.01	40.80 ± 13.06	39.56 ± 12.78	46.55 ± 13.99	< 0.001
Highest WBC (K/µL)	14.36 ± 12.03	14.41 ± 9.33	14.18 ± 14.84	16.73 ± 24.00	0.010
Lowest potassium (mEq/L)	3.96 ± 0.58	3.96 ± 0.57	3.96 ± 0.59	3.96 ± 0.66	0.941
Lowest Mg (mg/dL)	2.0 ± 0.4	2.0 ± 0.4	2.0 ± 0.4	2.0 ± 0.5	0.921
Highest glucose (mg/dL)	162.43 ± 89.17	157.88 ± 87.06	167.58 ± 89.44	212.01 ± 126.03	< 0.001
Lowest hemoglobin (g/dL)	10.02 ± 2.19	9.80 ± 2.04	10.44 ± 2.35	9.12 ± 2.38	< 0.001
Highest BUN (mmol/L)	32.72 ± 24.32	30.67 ± 22.64	35.42 ± 26.09	47.33 ± 31.18	< 0.001
Highest creatinine (mg/dL)	1.61 ± 1.46	1.53 ± 1.39	1.69 ± 1.48	2.75 ± 2.32	< 0.001
SC (mg/dL)	8.59 ± 0.78	8.15 ± 0.45	9.20 ± 0.36	11.28 ± 1.68	< 0.001
Highest heart rate (bpm)	107.70 ± 25.00	107.92 ± 24.96	106.98 ± 25.00	114.45 ± 25.40	< 0.001

### Associations between SC levels and patient outcomes

Table 1 details the results of the univariate analysis of risk factors related to in-hospital mortality among patients with critical AF, expressed as HRs and 95% CIs. Factors such as age, sex, hypertension, MI, OSAS, SOFA score, SAPSII score, WBC count, potassium, glucose, hemoglobin, blood urea nitrogen (BUN), creatinine, and heart rate were significantly associated with in-hospital mortality (*P* < 0.05). Conversely, variables like SC (*P* = 0.136), heart failure, pneumonia, type 2 diabetes, and type 1 diabetes were not significantly associated with in-hospital mortality (Table 1).

In this study, the relationship between SC levels and mortality was not significant when analyzed as a continuous variable (Table 1). Consequently, we categorized SC as a categorical variable, dividing patients into three groups based on the normal range (8.8–10.4 mg/dl): SC < 8.8 mg/dl, 8.8 mg/dl ≤ SC < 10.4 mg/dl, and SC ≥ 10.4 mg/dl. We then constructed two models to assess the independent impact of SC on in-hospital mortality (Table 3). In the multivariate Cox regression analysis, after adjusting for potential confounding factors and using the normal SC group (8.8–10.4 mg/dl) as the baseline reference, the relationships between both the high SC group (≥ 10.4 mg/dl) and the low SC group (< 8.8 mg/dl) with mortality remained non-significant in both Model I and Model II (*P* > 0.05) (Table 3).

**Table 3:** Relationships between different SC levels and in-hospital mortality in different models. Notes: data are presented as HRs and 95% CIs. Model I: adjusted for age, sex, ethnicity. Model II: adjusted for age, sex, ethnicity, hypertension, MI, OSAS, SOFA, SAPS II, WBC, potassium, glucose, hemoglobin, BUN, creatinine, and heart rate.

SC	Non adjusted Model		Model I		Model II	
	HR (95% CI)	P value	HR (95% CI)	P value	HR (95% CI)	P value
SC (mg/dL)	0.95 (0.88 ~ 1.02)	0.136	0.94 (0.87 ~ 1.01)	0.113	0.98 (0.91 ~ 1.05)	0.573
< 8.8 mg/dL	1.06 (0.93 ~ 1.20)	0.399	1.08 (0.95 ~ 1.23)	0.250	1.05 (0.92 ~ 1.20)	0.500
8.8 ~ 10.4 mg/dL	Reference		Reference		Reference	
≥ 10.4 mg/dL	0.99 (0.69 ~ 1.43)	0.964	1.09 (0.75 ~ 1.56)	0.658	1.06 (0.73 ~ 1.53)	0.769

However, the effect sizes observed in the multivariate Cox regression analysis indicated a pattern: the effect size for the high SC group (≥ 10.4 mg/dl) was 1.09 (HR: 1.09, 95% CI 0.75–1.56) in Model I and 1.06 (HR: 1.06, 95% CI 0.73–1.53) in Model II; the effect size for the low SC group (< 8.8 mg/dl) was 1.08 (HR: 1.08, 95% CI 0.95–1.23) in Model I and 1.05 (HR: 1.05, 95% CI 0.92–1.20) in Model II (Table 3). Using the normal SC group as the baseline reference, both high and low SC groups were associated with a higher risk of mortality (Table 3). These results suggest that the association between SC levels and in-hospital mortality in critically ill patients with AF may be nonlinear.

### The nonlinear relationship and sensitivity analysis

**Fig. 2:**
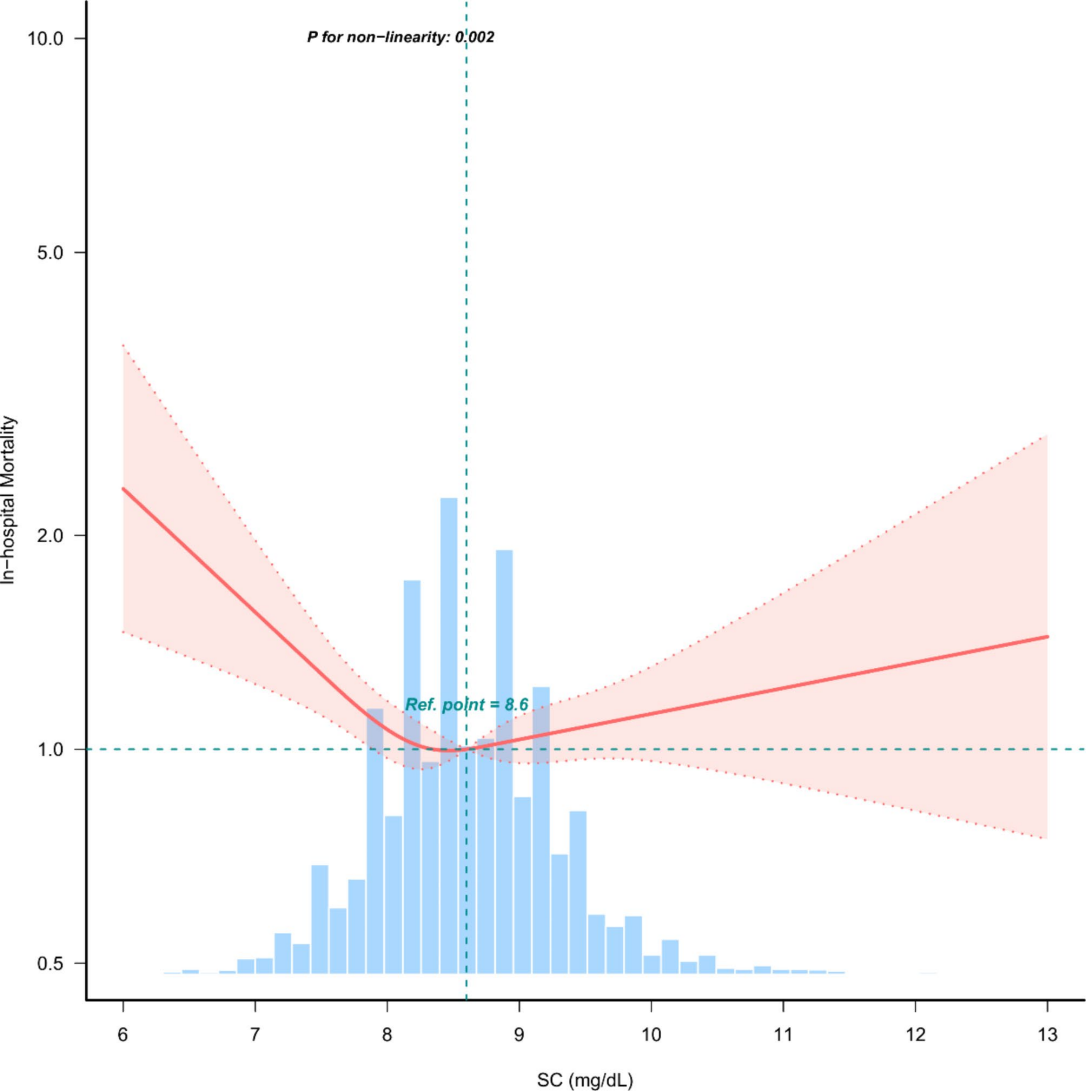
The nonlinear relationship between SC and in-hospital mortality in patients with AF in the ICU. Model II was adjusted for all covariates (SC was at the upper limit of 99.9%). AF: atrial fibrillation.

Through multivariate Cox regression analysis and smooth curve fitting, we determined that the relationship between SC levels and in-hospital mortality among AF patients in the ICU was nonlinear, exhibiting a “U”-shaped curve (Fig. 2). Data were analyzed using a piecewise multivariate Cox regression model with two distinct slopes. The P-value for the likelihood ratio test was less than 0.001 (Table 4), justifying the adoption of a bipartite model to elucidate the association between SC levels and ICU admission mortality in patients with severe AF. We identified an inflection point at approximately 8.56 mg/dL. Threshold effect analysis of SC levels and in-hospital mortality in patients with AF using Cox regression models. The threshold effect analysis results were significant in the non-adjusted model, Model I, and Model II (*P* < 0.05) (Table 4). On either side of this inflection point, the relationship between SC levels and in-hospital mortality differed significantly. To the left of the inflection point, the HR was 0.79 (95% CI 0.67–0.94, *P* = 0.006), indicating a 21% decrease in in-hospital mortality for each 1 mg/dL increase in SC. Conversely, to the right of the inflection point, the HR was 1.12 (95% CI 1.01–1.25, *P* = 0.029), corresponding to a 12% increase in mortality for each incremental mg/dL (Table 4).

**Table 4:** Threshold effect analysis of SC levels and in-hospital mortality in patients with AF using Cox regression models. Notes: data are presented as HRs and 95% CIs. Model I: adjusted for age, sex, and ethnicity. Model II: adjusted for age, sex, ethnicity, hypertension, MI, OSAS, SOFA, SAPS II, WBC, potassium, glucose, hemoglobin, BUN, creatinine, and heart rate.

	Non adjusted Model			Model I			Model II		
	HR	95% CI	P value	HR	95% CI	P value	HR	95% CI	P value
Turning point (mg/dL)	8.56			8.56			8.56		
SC < 8.56 mg/dL	0.69	0.59 ~ 0.82	< 0.001	0.67	0.57 ~ 0.79	< 0.001	0.79	0.67 ~ 0.94	0.006
SC ≥ 8.56 mg/dL	1.11	1.00 ~ 1.22	0.042	1.12	1.01 ~ 1.23	0.027	1.12	1.01 ~ 1.25	0.029

## Discussion

Previous studies have demonstrated the correlation between SC abnormalities and in-hospital mortality^8,23^. However, our findings indicate that SC levels, analyzed both as a continuous and categorical variable through univariate and multivariate Cox regression analyses, were not significantly associated with in-hospital mortality among critically ill patients with AF (*P* > 0.05). This discrepancy may stem from the nonlinear relationship observed between SC levels and in-hospital mortality in this patient group, a pattern corroborated by other studies investigating nonlinear dynamics^24,25^. Jianfei Hou et al. demonstrated that SC levels exhibit a nonlinear relationship with the risk of cardiac arrest in stroke patients, with an inflection point within the range of 8.2–10.4 mg/dL^26^. Similarly, James L. Lewis III reported the normal range for SC levels as 8.8–10.4 mg/dL (2.20–2.60 mmol/L)^27^. Considering that ICU patients with AF are at high risk for stroke, we selected the 8.8–10.4 mg/dL range for SC grouping. This decision is supported by both the findings from similar research and the normal SC range. Although stroke patients and AF patients are not identical, they share significant overlapping cardiovascular risk factors, particularly with regard to the development of complications like stroke, which validates the relevance of this SC range for our study population. Thus, the grouping of SC levels in our study is consistent with both physiological norms and findings from previous studies. In our analysis, using the normocalcemic group (8.8–10.4 mg/dL) as the reference, we observed increased mortality risks in both hypo- and hypercalcemic groups, suggesting a potential nonlinear relationship. This hypothesis was further supported by smooth curve fitting and sensitivity analysis. The result of P for nonlinearity: 0.002 indicates that the relationship between SC levels and in-hospital mortality among ICU patients with AF is nonlinear (Fig. 2). This statistically significant P-value suggests that a simple linear model may not fully capture the complexity of this association, further confirming the relationship may follow a “U”-shaped nonlinear trend.

In the context of complex data analysis and classification, recent advancements such as the polynomial model for protein acetylation sites^28^, the voting transfer model for oral microorganism function proteins^29^, the deep forest approach for Golgi protein classification^30^, and the application of machine learning methods in cardiovascular disease studies^31^ demonstrate the growing sophistication of techniques used to derive insights from intricate biological datasets. These studies highlight the potential benefits of employing advanced analytical methods, similar to those utilized in our analysis of SClevels and in-hospital mortality.

In recent years, the prevalence of nonlinear relationships in mortality predictor studies has increased^32–37^. Notably, several of these investigations have identified a “U-shaped” curve between predictive factors and mortality^38–43^. Our study confirms the nonlinear “U-shaped” relationship between SC levels and in-hospital mortality among ICU patients with AF. Using a smoothing curve, we identified an inflection point for SC levels at 8.56 mg/dL, with distinct relationships between SC levels and in-hospital mortality on either side of this threshold. Below the inflection point (SC < 8.56 mg/dL), the HR was 0.79 (95% CI 0.67–0.94, *P* = 0.006), indicating that each 1 mg/dL increase in SC was associated with a 21% reduction in in-hospital mortality. Conversely, above the inflection point (SC > 8.56 mg/dL), the HR was 1.12 (95% CI 1.01–1.25, *P* = 0.029), suggesting that each 1 mg/dL increase in SC was associated with a 12% increase in mortality. These findings imply that SC levels below 8.56 mg/dL are associated with a higher risk of in-hospital mortality, while SC levels above this point are linked to a relatively smaller increase in mortality risk. Although the HR of 1.12 for SC levels above the inflection point is statistically significant, its proximity to 1.0 suggests limited clinical significance. Similar trends were observed in Model I and the unadjusted model (Table 4), further supporting the idea that the statistically significant increase in risk may lack substantial clinical relevance.

Therefore, in our study, SC levels were identified as an independent prognostic factor for in-hospital mortality among ICU patients with AF, with lower SC levels showing a more pronounced clinical impact. Figure 2 visually represents this relationship. Similar observations have been noted in other populations^44–46^. For example, a UK study found that among patients with osteoarthritis, SC levels were an independent prognostic factor for CVD-related mortality. Lower SC levels (HR 2.06, 95% CI 1.02–4.14) had a more significant clinical impact compared to higher SC levels (HR 1.55, 95% CI 1.05–2.29)^47^. The study also reported that elevated SC levels were associated with an increased risk of all-cause mortality (HR 1.33, 95% CI 1.11–1.59), although the HR approached 1, suggesting limited clinical significance^47^. In related studies, higher SC levels have shown greater prognostic significance in cancer populations^48,49^. For instance, in critically ill patients with multiple myeloma, elevated SC levels (HR 2.104, 95% CI 1.069–4.142, *P* < 0.05) were associated with a significantly increased risk of in-hospital mortality compared to lower SC levels (HR 0.270, 95% CI 0.106–0.687, *P* < 0.05)^50^. The HR values in these cancer populations were distinctly greater than 1, highlighting the strong clinical impact of elevated SC levels. However, our study focuses on patients with AF rather than cancer patients, which may explain why the HR for SC levels above the inflection point in our analysis is closer to 1. This suggests that while elevated SC levels are statistically significant, their clinical impact may be less pronounced in the AF population compared to other conditions like cancer. In the treatment of ICU patients with AF, clinicians should closely monitor SC levels, with a focus on maintaining them around 8.56 mg/dL. When SC levels fall below 8.56 mg/dL, timely supplementation should be administered to increase SC levels, while levels above 8.56 mg/dL should prompt measures to reduce them. It is particularly important to note that patients with SC levels below 8.56 mg/dL face a higher risk of mortality^51^. Therefore, precise regulation of SC levels can help reduce in-hospital mortality and improve outcomes for patients with AF. This strategy holds significant clinical value in mitigating the risk of death.

The SAPS II is a widely used prognostic scoring model in the ICU^52^ that analyzes the severity of severe disease in patients. Furthermore, throughout the evolution of modern medicine, the SOFA score has consistently been considered as a trustworthy predictor of critical medicine^53^. Since its approval, the SOFA score has been utilized in a variety of contexts, including medical, trauma, surgical, cardiac, and neurological ICUs. The SOFA score has been demonstrated to be a good predictor of death, and the literature supports the SOFA score’s capacity to properly identify survivors from non-survivors at admission^54^. The SOFA score is now used to evaluate organ dysfunction in a variety of ICU settings, including medical, surgical, cardiac, neurological, transplant, respiratory care and step down units^55^. The key distinction between the two scores is that the SOFA score is primarily used for continuous monitoring of the severity and progression of organ failure, helping to track dynamic changes in the patient’s condition. In contrast, the SAPS II score focuses on assessing the patient’s physiological state at the time of ICU admission and provides a one-time prediction of mortality risk. Therefore, in our study, we corrected the SOFA and SAPS II scores for the illness condition of patients with AF. In our study, we corrected for both SOFA and SAPS II scores to account for the illness severity in AF patients. By using both, we were able to consider the severity of illness at admission (SAPS II) and track the progression of organ failure over the course of ICU treatment (SOFA), providing a comprehensive assessment of patient outcomes.

The study’s use of advanced statistical tools to assess the nonlinear relationship between SC levels and in-hospital mortality, rather than assuming a simple linear relationship, provides a more nuanced understanding of the data. This approach is superior because it captures the complexities of how SC levels impact mortality, identifying inflection points that linear models might miss, thereby offering greater clinical relevance and accuracy in guiding ICU physicians’ decisions. Two sensitivity analyses were done using SC levels. Furthermore, we used smooth curve fitting to determine the inflection point rather than manual grouping. These findings may lead ICU physicians to monitor SC levels when treating AF.

These findings should be interpreted in light of the following limitations. First, as a retrospective study, our use of administrative diagnosis codes to identify AF introduces a risk of misclassification, despite using the first diagnosis sequence. This could potentially lead to misleading associations. Second, our focus was on assessing the survival prognosis of ICU patients with AF, and it remains uncertain whether the identified inflection point applies to all AF patients or across different settings and time periods. Third, due to the nature of the simulation database, some critical variables, such as disease status and treatment details, may be missing. Nonetheless, we employed severity scoring systems, specifically the SOFA and SAPS II scores, to evaluate the severity and comorbidities associated with AF. Fourth, while the MIMIC-IV database is a reliable and comprehensive resource, its limitations must be acknowledged. The database relies on retrospective data collection, which can introduce biases related to data recording practices and missing information. Additionally, although MIMIC-IV provides extensive clinical data, it may not cover all relevant variables or reflect current clinical practices. These factors should be considered when interpreting the results and applying them to broader clinical contexts. Finally, given that this is a single-institution study, selection bias may also be a concern.

## Conclusions

In conclusion, our study found that SC levels are an independent prognostic factor for in-hospital mortality among ICU patients with AF. There is a nonlinear association between SC levels and in-hospital mortality, with the lowest mortality risk observed around an SC level of 8.56 mg/dL. SC levels below this inflection point are associated with a more pronounced clinical impact. Therefore, in the treatment of ICU patients with AF, clinicians should closely monitor SC levels, with a focus on maintaining them around 8.56 mg/dL. However, further prospective cohort studies are needed to confirm the relationship between SC levels and AF mortality.

## Data Availability

The data in the article can be obtained from the mimic-IV database (https://mimic.physionet.org/).
